# Small-quantity lipid-based nutrient supplements, with or without added zinc, do not cause excessive fat deposition in Burkinabe children: results from a cluster-randomized community trial

**DOI:** 10.1007/s00394-022-02936-6

**Published:** 2022-07-13

**Authors:** Souheila Abbeddou, Elizabeth Yakes Jimenez, Sonja Y. Hess, Jérome W. Somé, Jean Bosco Ouédraogo, Kenneth H. Brown

**Affiliations:** 1 Public Health Nutrition Unit, Department of Public Health and Primary Care, Ghent University, Campus UZ Gent, Corneel Heymanslaan 10, 9000 Ghent, Belgium; 2 Departments of Pediatrics and Internal Medicine and College of Population Health, University of New Mexico Health Sciences Center, Albuquerque, NM USA; 3 Research, International and Scientific Affairs, Academy of Nutrition and Dietetics, Chicago, IL USA; 4 Department of Nutrition and Institute for Global Nutrition, University of California, Davis, CA USA; 5 Institut de Recherche en Sciences de la Santé, Ouagadougou, Burkina Faso; 6 Institut de Recherche en Sciences de la Santé, Bobo-Dioulasso, Burkina Faso

**Keywords:** Child, Zinc, Body composition, Deuterium dilution technique, Fat-free mass, Fat mass, Small-quantity lipid-based nutrient supplements

## Abstract

**Purpose:**

Public health interventions to address stunting and wasting should be evaluated for possibly contributing to obesity risk. The present study tested the hypothesis that small-quantity lipid-based nutrient supplements (SQ-LNS) might increase fat deposition, and that additional zinc provided via SQ-LNS or in the form of dispersible tablets would increase fat-free mass (FFM) accretion.

**Methods:**

Using a two-stage, cluster-randomized trial design, 34 communities were randomly assigned to the intervention cohort (IC) or non-intervention cohort (NIC), and family compounds within the IC were randomly assigned to receive different amounts of zinc (0, 5 or 10 mg zinc) incorporated in SQ-LNS or 5 mg zinc in the form of dispersible tablets along with treatment for diarrhea, malaria and fever. Body composition was assessed in a subset of IC (*n* = 201) and NIC (*n* = 74) children at 9 and 18 months using the deuterium dilution method. A mixed linear model was used to examine average change in FFM and % fat mass (%FM) among intervention groups and by cohort.

**Results:**

Children in the IC had significantly greater change in FFM (Mean (95% Confidence Interval)) (1.57 (1.49, 1.64) kg) compared to the NIC (1.35 (1.23, 1.46) kg; *p* = 0.005). There were no significant differences in the change in %FM between the NIC and IC or among the intervention groups.

**Conclusion:**

SQ-LNS, along with morbidity treatment increased weight gain and FFM in young children from 9 to 18 months of age without increasing FM deposition. Additional zinc supplementation did not affect changes in FFM or %FM.

**Trial registration:**

The study was registered as a clinical trial with the US National Institute of Health (www.ClinicalTrials.gov; NCT00944281).

**Supplementary Information:**

The online version contains supplementary material available at 10.1007/s00394-022-02936-6.

## Introduction

Stunting, wasting, and underweight remain important public health problems among young children in low and middle-income countries (LMICs), where they impose an increased risk of childhood morbidity and mortality [1, 2], impair cognitive function and decrease adult economic productivity [3]. At the same time that undernutrition continues to plague children in LMICs, there has been a ten-fold increase in child and adolescent obesity over recent decades [4, 5], resulting in a situation referred to as “the double burden of malnutrition”. Obesity increases the risk of non-communicable diseases, including heart disease, type 2 diabetes, and some types of cancer [6]; and low birthweight and rapid physical growth during infancy may contribute to increased risks of obesity and associated non-communicable diseases in adulthood [7–9]. Thus, public health interventions to address childhood undernutrition must consider possible effects on the risk of obesity [10].

Lipid-based nutrient supplements (LNS) have been developed to improve child growth and reduce micronutrient deficiencies among children in LMICs [11–15]. In particular, small-quantity lipid-based nutrient supplements (SQ-LNS) provide an equivalent of 120 kcal daily, and approximately 50% of recommended vitamin and mineral daily intakes and essential fatty acids [16]. We have previously reported that providing SQ-LNS containing different amounts of zinc to children 9–18 months of age in southwestern Burkina Faso, together with community-based diagnosis and treatment of malaria and diarrhea, increased the children’s linear growth and weight gain and reduced the prevalence of stunting, wasting, anemia and iron deficiency compared to children in non-intervention communities, regardless of the zinc content of the supplements [11, 15]. Subsequent analyses indicated that this was likely due to the SQ-LNS component of the intervention [17]. In the present report, we describe the effects of the interventions on the body composition of a subset of children included in this trial.

Some, but not all, previous studies of zinc supplementation have found greater accrual of fat-free mass (FFM) among supplemented children [18]. For example, provision of 3 mg supplemental zinc daily to Peruvian infants for 6 months increased their FFM compared to children who did not receive additional zinc, but only in children with an initial length-for-age *z*-score less than the median of − 1.1 SD [19]. Similarly, supplementation of 30 or 50 mg zinc to rural Zimbabwean school children 11–17 years of age for 12 months during school days significantly increased arm muscle area for age *z*-score compared to the placebo group during the first three months of intervention, although this effect was not sustained once additional supplementary foods were distributed to all the children during the subsequent nine months [20].

To provide additional information on the potential effects of SQ-LNS containing different amounts of zinc on children’s body composition, we measured total body water (TBW) using the deuterium dilution method, and estimated the children’s fat mass (FM) and FFM at the beginning and end of a cluster-randomized, placebo-controlled supplementation trial (the International Lipid-based Nutrient Supplement-Zinc Trial, iLiNS-ZINC). The children in the intervention communities received SQ-LNS containing different amounts of zinc (0, 5 or 10 mg zinc) and dispersible tablets containing either 0 or 5 mg zinc from 9 to 18 months of age, along with treatment of identified episodes of malaria and oral rehydration salts for treatment of diarrhea. We also studied a separate set of children in non-intervention communities. The objectives of this sub-study were to determine: (1) the effects of the source of zinc (SQ-LNS or tablet) and the concentration of zinc in SQ-LNS on accrual of FM and FFM during the course of the intervention, and (2) the effects of the intervention package (regardless of zinc content) compared to non-intervention on accrual of FM and FFM during the course of the intervention.

## Subjects and methods

### Study site

The iLiNS-ZINC study took place from April 2010 to July 2012 in the Dandé Health District, a rural district in southwestern Burkina Faso, where childhood undernutrition is common and malaria is endemic [21]. The climate alternates between a rainy season from May to September and a dry season from October to April. Agriculture is the main source of income for most households, although food insecurity is highly prevalent [22].

### Study design

The parent study has been described in detail in previous publications [11, 15, 23, 24]. This trial included two levels of randomization: (1) at the community level and (2) at the concession (extended family compound) level. In the first level-randomization, 34 communities accessible during the rainy season were stratified by health clinic catchment area and were then randomly allocated to intervention cohort (IC, 25 communities) or non-intervention cohort (NIC, 9 communities), in such a way as to ensure balanced cohorts with respect to population size, distance from a paved road, and distance from the main city of Bobo-Dioulasso. In the intervention communities, concessions were randomly allocated to the intervention groups following a pre-generated randomization list prepared by the study statistician. Children in the IC were assigned to one of four treatment groups at the level of the concession to reduce the risk of cross-contamination within the family compound through food sharing. The investigators, field staff, study statistician, and all participants were blinded to the IC groups during the trial and initial phases of data analysis.

### Participants and intervention

**Fig. 1:**
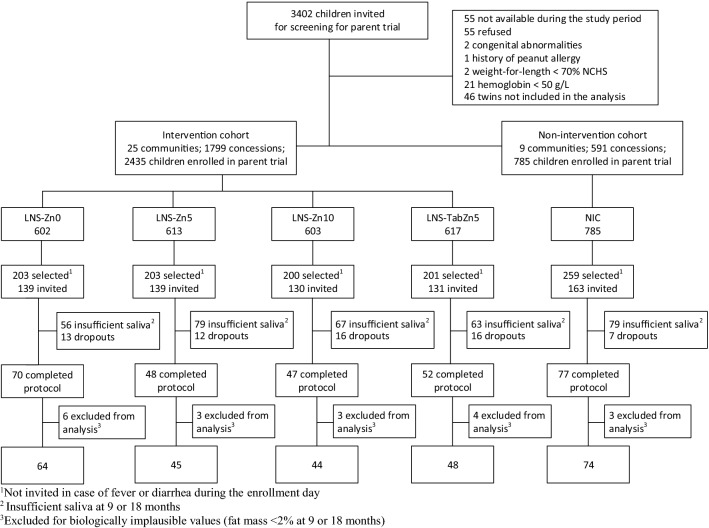
Flow diagram of participants in the body composition sub-study

A total of 3220 children 9–10 months of age were enrolled in the parent study (Fig. 1). Of these, 2435 children were included in the IC and 785 in the NIC. Children were considered eligible if they were permanent residents of the study area and their caregivers planned to be available during the nine-month study period and were willing to accept weekly home visits for morbidity assessments. Children were not enrolled in the study if their hemoglobin concentration (Hb) was < 50 g/L, weight-for-length was < 70th percentile of the National Center for Health Statistics/World Health Organization (NCHS/WHO) growth reference [25], or they had any illness warranting hospital referral or potentially interfering with growth [11, 15, 23].

Children in the IC were assigned to receive one of the following sets of daily supplements from 9 to 18 months of age: (1) SQ-LNS with no added zinc, and placebo tablet (LNS-Zn0); (2) SQ-LNS with 5 mg zinc, and placebo tablet (LNS-Zn5); (3) SQ-LNS with 10 mg zinc, and placebo tablet (LNS-Zn10); or (4) SQ-LNS with no added zinc, and 5 mg zinc tablet (LNS-TabZn5). More information on the nutrient composition of SQ-LNSs is given elsewhere [11]. All the study products were developed for the iLiNS project [16], and provided by Nutriset SAS (Malaunay, France). Supplementation of children in the IC started the day after all baseline data were collected, including the deuterium dilution study of body composition described below. Children in the NIC (*N* = 785) did not receive SQ-LNS or tablets from 9 to 18 months of age, but received SQ-LNS with 10 mg zinc for a 9-month period after the final saliva samples were collected.

Caregivers in the IC communities were instructed to administer 20 g SQ-LNS per day in two separate servings, preferably mixed in a small portion of the child’s meal, and to give the dispersible (zinc or placebo) tablet once daily at least 30 min away from meals to enhance zinc absorption. Adherence to both forms of supplements was assessed by obtaining information from maternal reports on daily administration and consumption of the supplements, and by collecting any remaining SQ-LNS, tablets and empty packages to estimate the disappearance rate each week [24]. At enrollment and throughout the study, the caregivers were advised to continue breastfeeding and to feed diverse local foods to the child.

At baseline, all affected children were treated for anemia, fever, malaria, and reported diarrhea following the national health guidelines in Burkina Faso [23]. Hb concentration in capillary blood was measured by Hemocue (Hemocue 201+, HemoCue AB, Ängelholm, Sweden). Children with Hb < 80 g/L received iron supplements (ferrous sulfate, 2–6 mg iron/kg body weight/d for 30 days, depending on the anemia severity) and 200 mg mebendazole/d for 3 days to treat possible helminthic infections. All children were screened for malaria parasites using a rapid diagnostic test (RDT, histidine-rich protein II; SD BIOLINE Malaria Ag P.F/Pan, Standard Diagnostics, Inc, Kyonggi-do, Korea). Those with a positive RDT received malaria treatment for three days (amodiaquine-artesunate, 1 tablet/d) and an antipyretic (paracetamol, 1/2 tablet three times daily for 3 days), and children with confirmed fever and a negative RDT and no other specific disease symptoms received paracetamol for three days. Children with reported diarrhea were given oral rehydration salts (ORS: 1 sachet/d for 4 days).

### Anthropometric, socioeconomic, dietary and morbidity data collection

Weight and recumbent length were measured in duplicate in all children at baseline and at ~ 18 months of age. Length was measured to the nearest 0.1 cm using a portable length board (Model 417, Seca, Hamburg, Germany), and weight was assessed with 50 g precision using an electronic balance (Model 383, Seca, Hamburg, Germany). If length measurements differed by > 0.5 cm or weight measurements differed by > 0.1 kg, a third measurement was completed and the average of the two closest values was used in the analysis.

Demographic and socioeconomic data were collected via questionnaire within 15 days of enrollment. Household food insecurity was assessed via the Household Food Insecurity Access Scale (HFIAS) [26]. At 9 and 18 months, dietary intake data and information on breastfeeding practices were obtained using adapted 24-h and 7-day food frequency questionnaires [27].

After enrollment, children in the IC were visited weekly by trained field agents who delivered the supplements and collected data on the children’s general health status, appetite and morbidity symptoms [23]. Treatment was provided in the case of reported diarrhea, reported or confirmed fever, and confirmed malaria based on positive RDT, as described above. Caregivers in the IC were advised to continue the previously assigned preventive supplementation regimens during illness. When any clinical danger sign, episode of diarrhea or malaria with complications, or signs of lower respiratory tract infection were reported, the child was referred to the local health clinic.

### Biochemistry subgroup

This paper reports on data obtained from a randomly selected subgroup of children who were included in the “biochemistry subgroup” (Fig. 1), as previously described [15] and who successfully provided saliva samples at both baseline and endline. Only one child from each concession was eligible to be included in the biochemistry subgroup to avoid reduced accuracy of estimation due to intra-cluster correlation. Among the selected children, those who had fever or reported diarrhea on the day of enrollment or on the day of body composition evaluation were not invited to the biochemistry assessment day or excluded on that day.

### Deuterium dose administration and saliva sample collection

Body composition was assessed using the deuterium dilution technique [28, 29]. We provided a constant dose of 4.0 g deuterium oxide (D_2_O, 99.8 atom % ^2^H, Cambridge Isotope Laboratory Inc., Andover, USA) for each assessment to achieve a concentration of > 600 mg D_2_O/kg saliva, as recommended for analyses using Fourier-transform infrared spectrometry (FTIR). This dose was confirmed to be adequate during pilot studies of ten children, seven male and three female, 13–23 months of age with body weights ranging from 10 to 12 kg and WAZ ranging from − 0.40 SD to 1.32 SD.

The pre-weighed doses of the deuterium tracer were prepared in the laboratory of the Institut de Recherche en Sciences de la Santé (IRSS, Bobo Dioulasso), using an analytical balance sensitive to 0.0001 g (Explorer Pro EP114C, Ohaus Corp., Switzerland) that was used only for this purpose. The tracer doses were placed in narrow-mouth bottles (capacity 8 mL, Thermo Scientific Nalgene, NY), sealed with parafilm to reduce the risk of evaporation, and stored in a refrigerator (4 °C) until used within the same week of their preparation. On the day of administration to the child, deuterium doses were transported to the field site in a cooler.

On the test day, the protocol was explained to the child’s caregiver. The child was then weighed in duplicate using an electronic scale with 5 g or 10 g graduation, depending on the actual weight of the infant (Model 334, Seca, Hamburg, Germany). Once the child’s weight was recorded, the first saliva sample was collected using small cotton ball(s). The saliva saturated cotton ball(s) were then inserted into a 10 mL-disposable syringe (Elite Medical (Nanjing) Co. LTD, China) and the saliva was expressed into a cryotube (3.6 mL, Nunc™ A/S, Denmark) and placed into a zip-lock bag and stored in a cooler (4–8 °C) for transport the same day to the IRSS laboratory, where they were stored at − 20 °C until the time of analysis. The D_2_O dose was mixed with a sweet strawberry-flavored syrup before oral administration, and small volumes of the diluted syrup were used to rinse the bottle twice to ensure administration of the full tracer dose. Children were allowed to breastfeed at will during the equilibration period; the amount of breastmilk consumed at each feeding episode was measured using test weighing. All children were also provided with a standardized snack (infant porridge). All foods and beverages consumed by the child were weighed before and after consumption using a portable kitchen balance. At both 2.5 and 3 h after administration of the D_2_0 dose, two post-dose saliva samples were collected and processed as described above for the baseline samples. Both samples collected at 2.5 and 3 h were analyzed in duplicate and the mean was used in data analyses as described in the next section. Studies were discontinued if the deuterium dose was not fully administered to the child (e.g., the child vomited or drooled some of the dose) or the amount of saliva obtained at any time point was insufficient (< 2 mL). Of the 702 infants invited to participate in the body composition assessments, 294 asymptomatic children provided adequate pre- and post-dose saliva samples at both 9 and 18 months of age (Fig. 1). Results are presented herein only for children who provided sufficient saliva samples at both 9 and 18 months of age for analysis of TBW. Data from 19 children were excluded from the final analyses because of implausibly low values (< 2%) for % fat mass (%FM), resulting in a final analytic sample of 275 children (Fig. 1).

### Laboratory analyses and calculation of TBW

Isotopic enrichment of saliva specimens was measured by FTIR (Shimadzu FTIR, Model 8400S, Shimadzu, Tokyo, Japan) using validated protocols recommended by the International Atomic Energy Agency (IAEA) [28, 29]. All samples were analyzed in duplicate [except in three cases where the volume was insufficient]. The coefficients of variation (CV) for the duplicate samples obtained at a single time point did not exceed 1%. If the CV of the mean of the four specimens analyzed for the two post-dose time points exceeded 5%, the most extreme value was eliminated, and the final post-dose value was calculated based on the mean of the remaining three values.

Deuterium concentrations of the pre-dose and the four post-dose samples (2.5 and 3 h) were determined using a calibration curve, and D_2_O enrichment was calculated by subtracting the pre-dose value from the final post-dose value. TBW in kg was calculated from the dilution of the deuterium tracer using the equation: TBW (kg) = dilution volume (Vol_D_)/1.041; where Vol_D_ = dose of D_2_O (g) administered to the child divided by the enrichment of D_2_O in the post-dose sample (mg/kg) [28]. Deuterium oxide overestimates TBW by 1.041 times, and therefore, to correct for the non-exchange of deuterium in the body, the TBW measurement was divided by 1.041. Water intake (from breastmilk or other foods/fluids) during the equilibration period was subtracted from the calculated TBW. FFM was calculated from TBW using a sex- and weight-specific hydration factor [30]. FM was then derived by subtracting the FFM from the total body weight and expressed either in kg or as a percent of body weight (%FM).

### Inflammation indicators

Acute phase proteins [C-reactive protein (CRP) and α-1-acid glycoprotein (AGP)], were analyzed by ELISA (DBS-Tech in Willstaett, Germany) in plasma samples which were collected from children on the same day as the body composition assessment, as described in details elsewhere [15, 31].

### Sample size for the body composition study

The sample size estimate for the body composition assessment was based on the number of children needed to detect differences in FFM with an effect size of 0.6 SDs for group-wise comparisons among the five groups, with a significance of *p* ≤ 0.05 and power ≥ 0.80. This anticipated effect size was based on the magnitude of effect found by Arsenault et al. [19]. The estimated sample size for the NIC was inflated for an assumed design effect of 1.5 due to the cluster sampling design, resulting in an estimated total sample requirement of 374 children in the 5 groups (68 in each of the 4 intervention groups and 102 in the NIC). This target sample size was increased to a total of 468 children in the 5 groups to allow for 20% attrition from 9 to 18 months.

### Data processing and statistical analysis

All outcomes were specified in the statistical analysis plan developed for the International Lipid-based Nutrient Supplements-ZINC (iLiNS-ZINC) Project (https://ilins.ucdavis.edu/). All statistical analyses were carried out using SAS software for Windows (9.3, SAS Institute, Cary, North Carolina). Descriptive statistics (means and standard deviation (SD), least squares mean (LSM) and standard error (SE) and proportions) were used to assess baseline information by study group and cohort, and to compare children who participated in the body composition assessment with those who were not included in these sub-studies. Variables were assessed for normality using the Shapiro–Wilk test.

Length-for-age (LAZ), weight-for-age (WAZ) and weight-for-length *z*-scores (WLZ) were calculated in relation to the WHO Child Growth Standards using SAS macros [32]. In addition to presenting the absolute data for FFM and FM, two indices of height-normalized body composition were calculated: the fat-free mass index (FFMI), calculated as FFM (kg)/length (m)^2^; and the fat mass index (FMI), calculated as FM (kg)/length (m)^2^ [33] for all children at 9 months and for 264 children for whom length data were available at 18 months of age. The indices were also plotted on Hattori charts, which are described in detail elsewhere [34]. In the Hattori chart, the x-axis represents FFMI and the y-axis FMI, with additional diagonal lines indicating body mass index (BMI; kg/m^2^) and %FM. Maternal BMI was calculated as maternal body weight in kg divided by the square of height in m, and classified according to the WHO standards as underweight (< 18.5 kg/m^2^), normal (18.5–24.99 kg/m^2^), or overweight/obese (≥ 25 kg/m^2^) [35].

Variables assessing breastfeeding practices, meal frequency, dietary diversity and consumption of nutrient-rich foods, including animal source foods, legumes, and fruits and vegetables were constructed based on the WHO indicators for assessing infant and young child feeding practices [36, 37]. The HFIAS Score was adjusted for season and year [26]. HFIAS score ranges between 0 and 27, with higher scores indicating greater food insecurity experienced by the household. Households were categorized using the Household Food Insecurity Access Prevalence (HFIAP) Status indicator into four levels of household food insecurity access: food secure, or mild, moderately and severely food insecure [26]. Using principal components analysis, we developed a wealth index based on baseline information on ownership of a set of assets, lighting source, drinking water supply, sanitation facilities, and flooring materials [38]; and we classified households into quartiles ranging from the poorest to the wealthiest households in the study population [38].

Elevated levels of CRP (> 5 mg/L) and/or AGP (> 1 g/L) were used as markers of inflammation [39]. Participants were categorized into four inflammation classes based on elevation of one or both acute phase proteins, or no inflammation [39]. Definitions of infectious diseases identified in IC children are reported in more detail elsewhere [11, 23]. Briefly, diarrhea was defined as caregiver report of three or more liquid or semi-liquid stools during a 24-h period. Fever was defined as any fever reported by the caregiver or elevated auricular temperature (> 37.5 °C), as measured by the field workers. Malaria was defined as the presence of reported or confirmed fever during the 24 h preceding the morbidity visit, associated with a positive RDT.

All models presented in this report use the body composition indicators after adjustment for breastmilk and food/fluids intake during the equilibration period, as recommended [28]. Body composition indicators and changes between baseline and at 18 months were compared by intervention group (four groups) and cohort using mixed models analysis of covariance (PROC MIXED). The models included a random effect of the community to account for intra-community correlation. Intervention group and cohort were used as the main effects. All the outcomes were adjusted for baseline values. Additionally, child sex and age at enrollment, maternal BMI (continuous), baseline child LAZ and WAZ (continuous), study season (rainy or dry), baseline child feeding practices including dietary diversity and consumption of animal source foods (all categorical), maternal education level and marital status were pre-specified and included as covariates. Intervention group means were compared post-hoc using least-square means with the Tukey–Kramer test.

Maternal BMI (continuous), baseline child LAZ (continuous variable and categorical variable as defined by the median), child sex, study season (rainy or dry), maternal education level and marital status were identified in advance of the analyses as possible effect modifiers of treatment group and cohort effects on the change in %FM and change in FFM between 9 and 18 months and interactions were examined using PROC MIXED.

### Ethical approvals

Ethical approval of the study protocol was provided by the Institutional Review Boards of the Centre Muraz in Bobo-Dioulasso (Burkina Faso) and the University of California, Davis (USA). Caregivers provided separate written, informed consents for their child’s participation in the parent study and for the collection of biological samples, including saliva, for the biochemical and body composition sub-studies.

## Results

### Baseline characteristics of participants in the body composition sub-study

Trial participants who were included in the body composition sub-study did not differ significantly from those who were not selected for the sub-study with regard to their mean age, feeding practices, morbidity during the course of the trial, or maternal BMI and education (Supplemental Table 1). At baseline, the children included in the sub-study weighed approximately 120 g more than those who were not included, and they had marginally greater WAZ and WLZ; but they did not differ significantly with regard to other anthropometrics (Supplemental Table 1).

The children in the sub-study had a mean age of 9.4 months at enrollment; 50.9% were boys and all were still breastfeeding (Supplemental Table 1). Overall, growth deficits were common, with 22.2% of children stunted (LAZ < − 2 SD), 28.7% underweight (WAZ < − 2 SD) and 14.5% wasted (WLZ < − 2 SD). Almost all children (91.6%) were anemic (Hb < 110 g/L) and 26.5% received iron supplementation and mebendazole at enrollment. Malaria and other infections were prevalent, with almost two-thirds of the children testing positive for malaria parasites at enrollment, and only 32% free of inflammation, based on normal CRP and AGP levels. Reported dietary intake data reflected poor feeding practices or food access. At 9 months, based on reported intake over the previous 24 h, 12.5% of children had not received solid or semi-solid foods, 74% did not meet the recommended minimum meal frequency, 75% had not received any animal source foods, and 84% did not meet minimum dietary diversity recommendations. Except for body weight and the dietary diversity score, none of the children’s baseline characteristics differed significantly by treatment group or cohort (Tables 1 and 2). Children in the LNS-TabZn5 group weighed (LSM ± SE) 534 g ± 192 g more than those in the LNS-Zn0 group (*p* = 0.047 for pair-wise comparison), and a greater percentage of the NIC children achieved the recommended minimum dietary diversity compared with the IC children (Table 1).

**Table 1:** Baseline child and maternal characteristics of participants included in the body composition sub-study, by study group and intervention cohort

	LNS-Zn0	LNS-Zn5	LNS-Zn10	LNS-TabZn5	*p* value^1,2,3^	IC	NIC	*p* value^1,2^
*N* (children who completed the protocol)	64	45	44	48		201	74	
*N* boys (%)	28 (43.7)	29 (64.4)	16 (36.4)	26 (54.2)	1.000	99 (49.2)	41 (55.4)	1.000
LAZ	− 1.37 ± 1.02	− 1.23 ± 1.15	− 0.97 ± 1.09	− 0.92 ± 1.11	0.131	− 1.14 ± 1.09	− 1.19 ± 1.16	0.713
WAZ	− 1.46 ± 1.08	− 1.31 ± 1.26	− 1.33 ± 1.02	− 0.95 ± 1.16	0.127	− 1.28 ± 1.14	− 1.37 ± 1.15	0.527
WLZ	− 0.98 ± 1.07	− 0.83 ± 1.10	− 1.04 ± 0.87	− 0.58 ± 1.12	0.136	− 0.85 ± 1.05	− 0.93 ± 0.98	0.549
*N* baseline iron supplementation (%)^4^	18 (28.1)	13 (28.9)	7 (15.9)	13 (27.1)	0.432	51 (25.4)	22 (29.7)	0.412
*N* baseline RDT positive (%)	44 (68.8)	26 (57.8)	29 (65.9)	29 (60.4)	0.618	128 (63.7)	40 (54.1)	0.237
Maternal body mass index (kg/m^2^)	20.6 ± 1.9	20.9 ± 2.0	21.0 ± 3.1	21.4 ± 2.3	0.353	20.9 ± 2.4	20.6 ± 2.3	0.298
Maternal education					0.788			0.398
No formal or informal education	40 (62.5)	29 (64.4)	27 (61.4)	26 (54.2)		122 (60.7)	51 (68.9)	
Any informal education and/or less than one year of formal education	19 (29.7)	11 (24.4)	13 (29.5)	14 (29.2)		57 (28.4)	15 (20.3)	
At least one year of formal education	5 (7.8)	5 (11.1)	4 (9.1)	8 (16.7)		22 (10.9)	8 (10.8)	
Baseline child feeding practices								
Child still breastfed (%)	64 (100)	45 (100)	44 (100)	48 (100)	1.000	201 (100)	74 (100)	1.000
Solid and semi-solid foods (%)	46 (86.8)	31 (91.2)	32 (91.4)	30 (85.7)	0.821	139 (88.5)	36 (83.7)	0.462
Minimum frequency of feeding meals (%)^5^	18 (34.0)	11 (32.3)	11 (31.4)	6 (17.1)	0.371	46 (29.3)	7 (16.3)	0.125
4 or more food groups (%)^6^	9 (14.1)	7 (15.6)	7 (15.9)	4 (8.3)	0.692	27 (13.4)	18 (24.3)	0.030
Animal-source food (%)^7^	16 (25.0)	10 (22.2)	10 (22.7)	11 (22.9)	0.342	47 (23.4)	22 (29.7)	0.998

*LAZ* length-for-age z-score, *WAZ* weight-for-age *z*-score, *WLZ* weight-for-length *z*-score

^1^Values presented are means ± SD, *n* (%)

^2^P-values are from mixed models for continuous variables, logistic regressions for binary variables and Chi square for polychotomous variables. All analyses were adjusted for the random effect of village

^3^Different letters indicate statistically significant differences between the groups at *p* < 0.05

^4^Children with Hb < 80 g/L received ferrous sulfate, 2–6 mg iron/kg body weight/d for 30 days, depending on the anemia severity and 200 mg mebendazole/d for three days to treat possible helminthic infections

^5^2 times per day for breastfed infants age 6–8 months of age, 3 times per day for infants 9–23 months of age, and 4 times per day for non-breastfed infants [37]

^6^Based on the WHO indicator, which sums intakes from seven food groups; a score of 4 or more is associated with higher nutrient density [36]

^7^Child consumed at least one animal-source food during the previous 24 h. Animal-source foods were defined as organ meats, other meat/poultry, fish, eggs and dairy products

**Table 2:** Body weight, length and body composition of children at 9 and 18 months of age, by study group and intervention (IC) or non-intervention (NIC) cohort

	LNS-Zn0	LNS-Zn5	LNS-Zn10	LNS-TabZn5	*p* value among 4 intervention groups^1,2,3^	IC	NIC	*p* value between cohorts^1,2^
At 9 months								
Body weight (kg)	7.32 ± 0.88^b^	7.60 ± 1.15^ab^	7.42 ± 0.95^ab^	7.84 ± 1.06^a^	0.038	7.53 ± 1.02	7.52 ± 1.05	0.931
Length (cm)	68.3 ± 2.4	69.0 ± 2.6	69.2 ± 2.7	69.6 ± 2.6	0.069	69.0 ± 2.6	69.0 ± 3.0	0.850
Total body water (kg)	4.56 ± 0.54	4.81 ± 0.62	4.70 ± 0.55	4.85 ± 0.65	0.047	4.71 ± 0.59	4.76 ± 0.59	0.681
Fat free mass (kg)	5.76 ± 0.68	6.07 ± 0.78	5.94 ± 0.69	6.12 ± 0.81	0.048	5.96 ± 0.75	6.01 ± 0.75	0.690
Fat mass (kg)	1.56 ± 0.59	1.53 ± 0.61	1.48 ± 0.51	1.72 ± 0.67	0.192	1.57 ± 0.60	1.51 ± 0.55	0.735
Fat mass (% body weight)	21.0 ± 6.4	19.7 ± 6.0	19.6 ± 5.4	21.6 ± 6.8	0.267	20.5 ± 6.2	19.7 ± 5.7	0.511
Fat free mass index (kg/m^2^)	12.3 ± 1.0	12.7 ± 1.1	12.4 ± 0.9	12.6 ± 1.3	0.160	12.5 ± 1.1	12.6 ± 0.9	0.629
Fat mass index (kg/m^2^)	3.3 ± 1.2	3.2 ± 1.1	3.1 ± 1.0	3.5 ± 1.3	0.203	3.3 ± 1.2	3.1 ± 1.1	0.564
At 18 months								
Body weight (kg)	9.05 ± 0.95	9.40 ± 1.13	9.26 ± 1.19	9.72 ± 1.05	0.536	9.33 ± 1.09	9.10 ± 1.17	0.002
Length (cm)	77.2 ± 2.5	78.1 ± 2.8	78.2 ± 3.1	78.6 ± 2.9	0.580	78.0 ± 2.8	77.3 ± 3.5	0.011
Total body water (kg)	5.71 ± 0.63	5.95 ± 0.68	5.89 ± 0.87	6.12 ± 0.64	0.498	5.90 ± 0.71	5.78 ± 0.63	0.005
Fat free mass (kg)	7.28 ± 0.80	7.58 ± 0.86	7.51 ± 1.11	7.80 ± 0.81	0.498	7.52 ± 0.91	7.36 ± 0.80	0.005
Fat mass (kg)	1.77 ± 0.56	1.82 ± 0.53	1.74 ± 0.49	1.92 ± 0.62	0.963	1.81 ± 0.55	1.74 ± 0.64	0.412
Fat mass (% body weight)	19.4 ± 5.2	19.2 ± 4.4	18.9 ± 4.6	19.6 ± 5.5	0.944	19.3 ± 4.9	18.7 ± 5.2	0.691
Fat free mass index (kg/m^2^)	12.3 ± 1.0	12.4 ± 0.9	12.2 ± 1.3	12.6 ± 0.9	0.527	12.4 ± 1.0	12.3 ± 0.9	0.175
Fat mass index (kg/m^2^)	2.9 ± 0.8	3.0 ± 0.8	2.9 ± 0.8	3.1 ± 1.0	0.974	3.0 ± 0.8	2.9 ± 1.0	0.539
Change from baseline to 18 months								
Body weight (kg)	1.72 ± 0.59	1.80 ± 0.67	1.84 ± 0.65	1.88 ± 0.64	0.536	1.80 ± 0.63	1.57 ± 0.55	0.002
Length (cm)	8.8 ± 1.4	9.1 ± 1.4	9.1 ± 1.3	9.0 ± 1.7	0.580	9.0 ± 1.4	8.4 ± 1.9	0.011
Total body water (kg)	1.16 ± 0.41	1.14 ± 0.40	1.19 ± 0.53	1.27 ± 0.43	0.498	1.19 ± 0.44	1.01 ± 0.39	0.005
Fat free mass (kg)	1.53 ± 0.52	1.51 ± 0.51	1.57 ± 0.68	1.67 ± 0.54	0.498	1.57 ± 0.56	1.35 ± 0.49	0.005
Fat mass (kg)	0.19 ± 0.46	0.29 ± 0.49	0.27 ± 0.60	0.20 ± 0.58	0.963	0.23 ± 0.53	0.23 ± 0.50	0.412
Fat mass (% body weight)	− 1.8 ± 5.2	− 0.5 ± 5.1	− 0.7 ± 6.5	− 2.0 ± 6.2	0.944	− 1.3 ± 5.7	− 1.0 ± 5.5	0.691
Fat free mass index (kg/m^2^)	− 0.01 ± 0.9	− 0.3 ± 0.8	− 0.1 ± 1.0	− 0.02 ± 1.0	0.527	− 0.1 ± 0.9	− 0.3 ± 1.0	0.175
Fat mass index (kg/m^2^)	− 0.4 ± 1.0	− 0.2 ± 0.9	− 0.2 ± 1.1	− 0.4 ± 1.1	0.974	− 0.3 ± 1.0	− 0.2 ± 0.9	0.539

^1^Adjusted means ± SD, all such values

^2^Values are adjusted for the random effect of the village, for the respective baseline values and for selected covariates including child sex and age at enrollment, maternal BMI (continuous), baseline child LAZ and WAZ (continuous), study season (rainy or dry), baseline child feeding practices including dietary diversity and consumption of animal source foods (all categorical), maternal education level and marital status using Proc MIXED

^3^Different letters indicate statistically significant differences between the groups at *p* < 0.05

The study population was rural and food insecure; 9% had severe food insecurity access and 31% had moderate food insecurity access. The mothers’ mean age was 26.9 ± 6.6 years, almost all were married (98.2%), and approximately two thirds had at least one additional child under 5 years of age. More than half the mothers had received no formal or informal education, and only ~ 10% received more than 1 year of formal schooling. The majority of mothers had normal BMI, but 16% were underweight and 6% were overweight/obese.

### Quantities of breastmilk, water and food intake during the tracer equilibration period

A total of 266 children consumed a mean of 79.8 ± 47.0 g breastmilk (range 5–370 g) during the tracer equilibration period at baseline and 262 children consumed 80.4 ± 50.6 g at 18 months of age (range 5–345 g). Children consumed total quantities of food and fluids ranging from 1 to 241 g (mean ± SD: 49.6 ± 38.7 g) at 9 months of age (*n* = 198) and 1–434 g (84.7 ± 66.4 g) at 18 months of age (*n* = 246).

### TBW, fat-free mass and fat mass at baseline

The children’s mean body weights and body composition at the time of the initial TBW study are shown by study group and cohort in Table 2. As noted above, children in group LNS-TabZn5 weighed slightly more than those in group LNS-Zn0, which was also reflected in differences in the respective groups’ TBW and FFM. However, there were no significant group-wise differences in FM, %FM, or FFM or FM indices, nor were there differences in any of the indicators by intervention cohort. The overall mean (CI 95%) TBW at baseline was 4.72 (4.66, 4.80) kg [range 3.42–6.55 kg], the mean FFM was 5.97 (5.88, 6.06) kg, the mean FM was 1.56 (1.49, 1.62) kg, and the mean %FM was 20.3 (19.6, 21.0) %. Boys had a slightly greater TBW than girls (4.92 (4.84, 5.02) kg vs. 4.52 (4.42, 4.62) kg, respectively, *p* < 0.0001), but there were no significant differences between boys and girls in FM or %FM.

### Effect of the intervention on the children’s final body composition

**Fig. 2:**
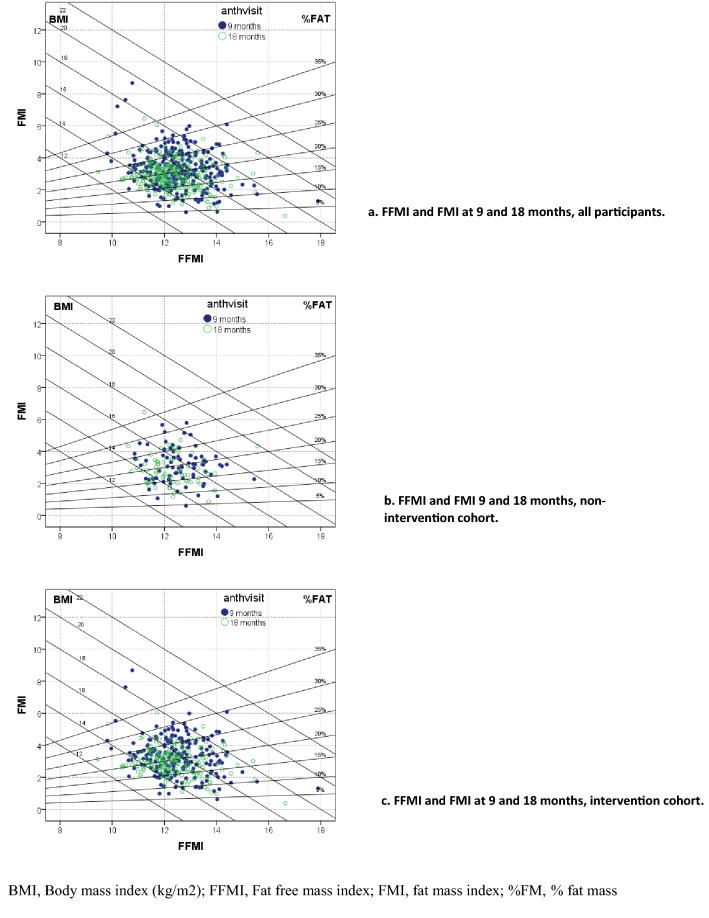
Fat free mass index (FFMI) and fat mass index (FMI) of children at 9 and 18 months of age (**a**), and by intervention cohort (**b**, **c**)

There were no differences in any of the body composition outcomes by study group within the intervention cohort (Table 2), so this presentation focuses on outcome differences between the intervention and non-intervention cohorts. Children in the IC gained more weight (1.8 (1.72, 1.89) vs 1.6 (1.45, 1.70) kg, *p* = 0.004), length (9.0 (8.81, 9.22) vs 8.4 (7.94, 8.84) cm, *p* = 0.005), TBW (1.19 (1.13, 1.25) vs 1.01 (0.92, 1.10) kg, *p* = 0.006) and FFM (1.57 (1.49, 1.64) vs 1.35 (1.23, 1.46) kg, *p* = 0.006) than those in the NIC. However, there were no significant differences in the accrual of FM, %FM, or FFM or FM indices by intervention cohort. At 18 months of age, the FFMI was ~ 0.2 kg/m^2^ lower (*p* = 0.002) and the FMI was ~ 0.3 kg/m^2^ lower (*p* < 0.0001) than at 9 months, indicating that the proportions of FFM and FM decreased slightly in relation to height over this period in both cohorts (Fig. 2).

### Other factors possibly affecting the change in fat and fat free mass in children

We examined the possible modifying effects of maternal BMI, child sex, baseline HAZ (both as a continuous and a categorical variable), study season, maternal education, and maternal marital status on the relationships between study group and intervention cohort and change in %FM and FFM, and we found no significant interactions.

## Discussion

Daily provision of SQ-LNS containing different amounts of zinc to young children from 9 to 18 months of age, along with morbidity treatment and periodic counseling on child feeding practices, had a positive impact on gains in body weight, length, TBW and FFM in young Burkinabe children, but did not affect the relative proportions of FM accrual compared with a non-intervention cohort, regardless of whether FM was expressed in absolute terms or in relation to height (as FMI). Both the FMI and FFMI decreased slightly over the age range of the study, which is consistent with observations in reference children [34]. The additional weight gain resulting from the intervention was comprised mostly of FFM, as expected for children of this age range [34]. There were no observed effects of the different doses of zinc, provided either in the SQ-LNS or as separate dispersible tablets, on changes in body composition.

Children participating in this study had lower TBW than reference populations reported in the literature [30, 40–44], as would be expected because of the study children’s lower body weights and WAZs. TBW of reference healthy children from high income countries range between 4.8 and 5.2 kg at 9 months, and from 6.1 to 6.6 kg at 18 months [28]. There are limited data on body composition of similar, non-acutely malnourished African children, but one community-based study of Gambian infants likewise found that their FFM and FM were lower than observed in UK infants, with or without adjustment for height [45]. By contrast, Owino et al. reported that FFM in Zambian children at 9 months of age (average 6.9 kg) was similar to reference values, but the Zambian children were heavier than the children in our study population (average WAZ > 0.10 SD compared to − 1.4 SD, respectively), and were from middle-income urban areas with better socio-economic status, food access and a lower burden of infectious diseases [46]. Birth weight, breastfeeding and other infant feeding practices, infectious disease burden and genetic and epigenetic factors may all contribute to differences in body composition at this age [45].

It is well established that infants and children with rapid weight gain trajectories, defined as an increase in WAZ greater than 0.67 SD over 4–24 months [8], are at highest risk for developing obesity and associated chronic diseases later in life [9, 47]. Despite the greater weight gains of the IC compared with the NIC in the present study, their WAZ only increased by 0.19 SD, so they would not be considered at high risk of obesity and related diseases. Similar to the current results, a study in rural Bangladeshi children 6–18 months of age found that provision of industrial or locally developed LNS, or blended complementary foods did not cause excessive fat deposition [48]. Notably, in that study the supplements increased the children’s WAZ by just 0.02 SD − 0.04 SD/month [49], which is also well below the cutoff considered to cause later obesity. Studies on the effect of lipid-based nutrient supplements on body composition are more available in the context of treatment of moderate acute malnutrition (MAM), where MAM children receive medium quantity-LNS (MQ-LNS) in a 12 week treatment. The two studies carried out in Burkina Faso and Mali found no adverse effects on FM accrual [50, 51].

Zinc is essential for lean tissue synthesis, either because of its direct incorporation into newly synthesized tissue [52] or its indirect effects on appetite and energy intake [19]. However, providing additional zinc had no impact on body composition in the present study. As reported previously, we also found that delivering additional zinc, either in SQ-LNS or as dispersible tablets, did not independently affect the children’s linear growth, weight gain, or PZC in this study. Possible explanations for this set of findings are poor adherence to the supplements or poor absorption or utilization of the additional zinc. Alternatively, the study participants may have been unresponsive in growth or body composition because they were not zinc deficient or other nutrient deficiencies or infections limited their responsiveness to additional zinc. We have previously reported that apparent adherence to supplementation varied according to how the information on adherence was obtained and the form of the supplement [24]. Whereas reported adherence and supplement disappearance rates were high for both SQ-LNS and tablets (~ 97%), observed consumption during 12-h, in-home dietary observations was considerably lower, especially for the tablets (~ 30%). Previous studies show that PZC almost always responds to zinc supplementation [53], so the lack of difference in PZC among those who were supplemented suggests that poor adherence may indeed have been an issue. On the other hand, children who received SQ-LNS finished the study with significantly greater serum ferritin and hemoglobin concentrations than those in the comparison group, indicating that the SQ-LNS was being consumed [15]. With regard to the children’s zinc status, 36% of the participants in the parent study had PZC < 65 μg/dL at baseline, which is the lower range of the PZC distribution in a presumably well-nourished population [54]. Although PZC can be useful for assessing a population’s risk of zinc deficiency [55], it does not necessarily predict functional responses to zinc interventions, so we remain uncertain about the zinc status of the study participants.

Our results regarding the LNS-TabZn5 group differ from some other zinc supplementation studies, where providing zinc in the form of tablet or syrup increased both linear growth and lean mass accretion relative to providing it via food. For example, zinc supplementation produced a greater increase in FFM (1.36 kg) among 6–8-month-old Peruvian children with initial mild-to-moderate stunting compared with providing zinc in a fortified porridge (0.95 kg; *p* = 0.02) or providing no additional zinc (0.95 kg; *p* = 0.04) [19]. Positive effects of zinc supplementation on FFM have also been found in pre-pubertal, short girls with sickle cell disease in the United States when FFM was estimated using skinfold-thickness but not when measured by dual-energy X-ray absorptiometry (DEXA) [56]; on TBW in premature infants in the Canaries [57]; on median triceps skinfold *Z*-score of 6–7-year old peri-urban Guatemalan children [58]; and on mid-arm muscle area of Guatemalan children [59]. In contrast to these results, a randomized controlled trial conducted in Zimbabwe found no effect of supplemental zinc (30 or 50 mg daily for one year) on lean body mass or weight gain in adolescent (ages 11–17 years) schoolchildren [20]. A recent systematic review that assessed the effect of zinc supplementation on body composition among children in community- or hospital-based settings from 14 high- and low- and middle-income countries showed inconsistent results [18]. Overall, zinc supplementation had a beneficial effect on FFM accretion among stunted [but not non-stunted] children. Heterogeneous results regarding the effect of zinc supplementation on body composition may be attributable to differences in study designs and supplementation doses, forms, and duration, but also to the different techniques used in assessing body composition and to different contextual and environmental factors.

Our study has several strengths. Body composition was assessed using the deuterium dilution method, a two-compartment method that offers high quality measurements that can be reasonably implemented in rural field settings. The laboratory at the IRSS, Bobo Dioulasso had been previously standardized in the context of a multi-laboratory comparison of FTIR analyses conducted by the International Atomic Energy Agency (The IAEA, unpublished report).

We also recognize some study limitations due to the challenges faced in the field. Repeated two ml saliva samples were collected successfully in just 79% of the desired sample size because of difficulties obtaining the desired specimen volumes from young children in this hot environment over the course of a field protocol that takes several hours. After our study was completed, a newer version of the FTIR spectrometer became available (Agilent 4500 Series FTIR), which can complete measurements with just 50 µL saliva. This could facilitate body composition assessment in young children in this and other similar settings in the future. Despite this limitation, our final sample size had adequate statistical power to detect differences in FM and FFM of as little as 0.48 SDs between IC and NIC, 0.87 SDs across the four zinc groups and 0.83 SDs across all five groups, so the limited sample size should not undermine our conclusions. Also, inherent in the calculation of FFM for underweight children is the assumption that undernourished children have the same hydration factor as non-malnourished individuals of the same age and sex. This assumption could lead to overestimation of the FFM [28]. In our case, we compared TBW directly to limit the bias that could be produced by the available models that do not adjust for nutritional status. There is a need to evaluate body composition of undernourished infants and young children with more sophisticated methods including a four-compartment technique in order to validate the hydration coefficients [60].

## Conclusions

Our study demonstrates that providing daily SQ-LNS along with morbidity treatment had a beneficial effect on weight gain and FFM gain from 9 to 18 months of age. The SQ-LNS did not alter the composition of weight gain and had no adverse effects on FM accretion. The present study did not find an effect of zinc supplementation, whether included in SQ-LNS or in the form of dispersible tablet, on body composition. Incomplete adherence to the supplements, poor absorption or utilization of the additional zinc, and other micronutrient deficiencies that are growth limiting may have contributed to the lack of effect of zinc on physical growth and FFM accretion in our study population.

## Supplementary Information

Below is the link to the electronic supplementary material.

Supplementary file1 (PDF 219 KB)

## Data Availability

Data used in this analysis are available at: https://osf.io/7dy8j/
