# Supplementary material for: Small-quantity lipid-based nutrient supplements, with or without added zinc, do not cause excessive fat deposition in Burkinabe children: results from a cluster-randomized community trial
Source: Eur J Nutr. 2022 Jul 13;61(8):4107–20. doi: 10.1007/s00394-022-02936-6 (PMC9596589; doi:10.1007/s00394-022-02936-6)
Supplement: Supplementary file 1 — Supplementary file1 (PDF 219 KB) [file 394_2022_2936_MOESM1_ESM.pdf]

Supplementary table 1: Child, maternal and household characteristics of study participants included in the body composition sub-study, compared with study participants not included in the body composition sub-study

	In subgroup combined	Not in subgroup	*p-value*[1,2]
N (children with 9 and 18 mo values)	275	2945	
Child age (mo)	9.4 ± 0.33	9.4 ± 0.35	0.204
N boys (%)	141 (51.1)	1479 (50.2)	0.803
Baseline weight (kg)	7.54 ± 1.02	7.42 ± 1.00	0.043
Baseline length (cm)	69.0 ± 2.7	68.8 ± 2.7	0.161
Baseline child LAZ (SD)	-1.15 ± 1.11	-1.21 ± 1.10	0.245
Baseline child WAZ (SD)	-1.30 ± 1.14	-1.43 ± 1.14	0.057
Baseline child WLZ (SD)	-0.87 ± 1.03	-1.00 ± 1.05	0.054
Hemoglobin concentration (g/L)	89.2 ± 14.9	89.3 ± 15.3	0.926
N Baseline iron supplementation (%)[3]	74 (26.8)	779 (26.5)	0.840
N Baseline RDT positive (%)	169 (61.2)	1809 (61.4)	0.992
Maternal body mass index (kg/m^2)	20.9 ± 2.3	20.9 ± 2.5	0.804
Maternal education			0.669
No formal or informal education	173 (62.7)	1746 (59.9)	
Any informal education and/or less than one year of formal education	73 (26.4)	854 (29.3)	
At least one year of formal education	30 (10.9)	313 (10.7)	
Baseline child feeding practices			
Child still breastfed (%)	276 (100)	2940 (99.97)	0.980
Animal source food[4]	69 (25.0)	646 (21.9)	0.303
Child morbidity during the intervention[5]			
Child malaria incidence (episodes per 100 child days at risk)[6]	0.61 ± 0.50	0.53 ± 0.50	0.843
Child fever incidence (episodes / 100 child-days at risk)[7]	1.46 ± 1.00	1.49 ± 1.13	0.465
Child diarrhea incidence (episodes per 100 child days at risk)[8]	0.96 ± 0.91	1.11 ± 1.04	0.076

LAZ, length-for-age z-score; WAZ, weight-for-age z-score; WLZ, weight-for-length z-score.

[1]Values presented are means ± SD, n (%).

[2]P-values are from mixed model for continuous variables, logistic regression for binary variables and Chi square for polychotomous variables. All analyses were adjusted for the random effect of village, and in morbidity outcomes, were controlled for over-dispersion.

[3]Children with Hb <80 g/L received ferrous sulfate, 2–6 mg iron/kg body weight/d for 30 days, depending on the anemia severity, and 200 mg mebendazole/d for three days to treat possible helminthic infections.

[4]Child consumed at least one animal-source food during the previous 24 hours.

[5]Morbidity outcomes include children in the IC who provided data on ≥30 days.

[6]An episode of malaria was defined as the presence of a new episode of reported or confirmed fever and a positive malaria RDT obtained >21 days after a previous treated malaria episode.

[7]An episode of fever was defined as the period starting the day the child first had fever preceded and followed by two days when the child did not have fever.

[8]An episode of diarrhea was defined as the period starting the day the child first had diarrhea preceded and followed by two diarrhea-free days.
